# The implication of plastid transcriptome analysis in petaloid monocotyledons: A case study of *Lilium lancifolium* (Liliaceae, Liliales)

**DOI:** 10.1038/s41598-019-43259-7

**Published:** 2019-04-30

**Authors:** Hoang Dang Khoa Do, Joo-Hwan Kim

**Affiliations:** Department of Life Science, Gachon University, Seongnam, Gyeonggi-do 13120 Korea

**Keywords:** Molecular evolution, Plant evolution

## Abstract

Transcriptome data provide useful information for studying the evolutionary history of angiosperms. Previously, different genomic events (i.e., duplication, deletion, and pseudogenization) were discovered in the plastid genome of Liliales; however, the effects of these events have not addressed because of the lack of transcriptome data. In this study, we completed the plastid genome (cpDNA) and generated transcriptome data of *Lilium lancifolium*. Consequently, the cpDNA of *L*. *lancifolium* is 152,479 bp in length, which consists of one large single copy (81,888 bp), one small single copy (17,607 bp), and two inverted repeat regions (26,544 bp). The comparative genomic analysis of newly sequenced cpDNA and transcriptome data revealed 90 RNA editing sites of which two positions are located in the rRNA coding region of *L*. *lancifolium*. A further check on the secondary structure of rRNA showed that RNA editing causes notable structural changes. Most of the RNA editing contents are C-to-U conversions, which result in nonsynonymous substitutions. Among coding regions, *ndh* genes have the highest number of RNA editing sites. Our study provided the first profiling of plastid transcriptome analyses in Liliales and fundamental information for further studies on post-transcription in this order as well as other petaloid monocotyledonous species.

## Introduction

In the genomic era, besides the nuclear, mitochondrial, and plastid genomes, transcriptome data also provide useful information for exploring the evolutionary history of angiosperms. Previously, different transcriptome studies of model plants by applying next-generation sequencing method (NGS) were reported, including *Arabidopsis thaliana*, rice, sugarcane, and so on^1–5^. These data added a deeper understanding of the gene expression and biological mechanisms that allow plants to survive and adapt to the environment. For example, transcriptome data revealed the mechanism that showed how sugarcane responded after being infected by bacteria or a virus^3,5^. Also, transcriptome data suggested genes that are responsible for drought and salinity tolerance in rice^4^. The transcriptome analysis was not only reported for model plants but also wild species. For instance, RNA editing sites were observed in *Spirodela polyrhiza* and *Phalaenopsis aphrodite* subsp *formosana*^6,7^. Also, the mechanism of cold responses in *Lilium lancifolium* was profiled from transcriptome data^8^. In plants, RNA editing resulted from the modification of nucleotide sequences. This process has a significant effect on gene expression because it can cause the presence of an internal stop codon^9–11^. In fact, the effect of RNA editing on the metabolism of plants was reported^12^. These studies suggested the necessity of transcriptome data in studying the evolution in angiosperms.

Liliales is a member of petaloid monocotyledons and consists of 10 families of 1558 taxa^13^. The plastid genomes of this order have been intensively studied, and different genomic events (i.e., inversion, deletion, duplication, and pseudogenization) were discovered^14–19^. For example, various stages of *rps16* deletion were recorded in the tribe Melanthieae (Melanthiaceae, Liliales)^19^. Also, duplication events were found in the *Paris * species^16,18^. Although genomic events were reported, their effects on the post-transcriptional process are unclear because of the lack of plastid transcriptome data in Liliales. Therefore, in this study, we conducted the first plastid transcriptome analysis in Liliales. First of all, *Lilium lancifolium* was selected as the target species. Then, complete plastid genome (cpDNA) of *L*. *lancifolium* was sequenced using Next-generation sequencing method. Based on the new RNA editing data, we test the hypothesis of whether the pseudogenization can be reversed in *L*. *lancifolium*. We also check the effect of RNA editing site on the secondary structure of rRNA.

## Results

### Plastid genome of *Lilium lancifolium*

**Figure 1:**
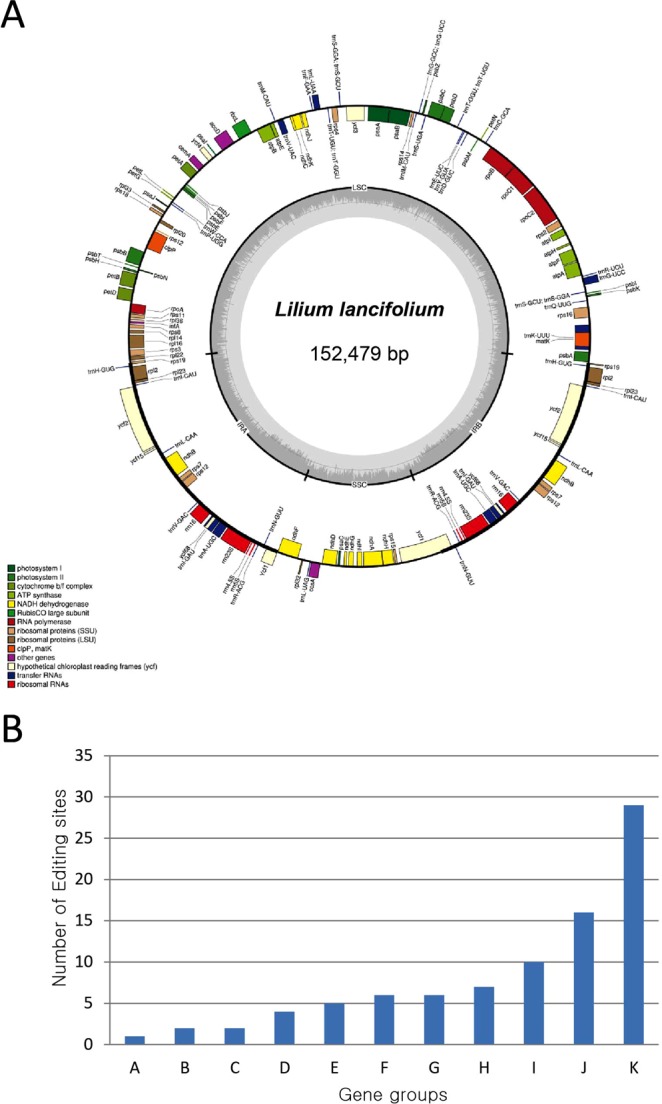
The map of plastid genome and number of RNA editing sites in different gene groups. (**A**) The map of plastid genome of *Lilium lancifolium*. Genes shown outside and inside of the outer circle are transcribed counter clockwise and clockwise, respectively. The dark gray area in the inner circle indicates the CG content of the chloroplast genome. The colors represent different groups of genes in cpDNA. LSC: Large single copy; SSC: small single copy; IRA: inverted repeat region A; IRB: inverted repeat region B. (**B**) The number of RNA editing sites in different gene groups. A: Rubisco; B: ATP dehydrogenase subunit P; C: Ribosomal RNAs; D: Cytochrome b6/f; E: Hypothetical proteins; F: ATP synthase; G: Miscellaneous proteins; H: Large and small subunit ribosomal proteins; I: Photosystem I and II; J: RNA polymerase; K: NADH oxidoreductase.

The complete plastid genome sequence of *L*. *lancifolium* (accession number MH177880; Fig. 1A) in this study is 152,479 bp in length and composed of a large single copy (LSC; 81,888 bp), a small single copy (SSC; 17,607 bp), and two inverted repeat regions (IR; 26,492 bp). In comparison with the previously completed cpDNA of *L*. *lancifolium* from China (accession number KY748297) and Korea (accession number KY940844), of which the length of cpDNA is identical (152,574 bp), the gene contents and orders are similar among the three individuals. However, the percentage of the identity of new cpDNA in *L*. *lancifolium* in this study is 99.8% and 99.9% compared to counterparts from Korea and China, respectively. Also, the translation initiation factor IF-1 (*infA*) gene was not annotated in previous data, but it was predicted as a pseudogene in this study because of the presence of internal stop codons within the coding region. Additionally, the different length of poly A sequence after start codon caused two types of *cemA* gene. The first type is functional *cemA* in *L*. *lancifolium* from China, of which 9-bp-poly A sequence was found. In contrast, the malfunctioning *cemA* was annotated in cpDNA from Korea counterparts, of which 10-bp and 11-bp-poly A sequences were found and caused internal stop codons in the coding region. The IGS regions among three cpDNA sequences of *L*. *lancifolium* showed a high similarity (over 95%), except the ISG regions of *petA-psbJ*, *ndhF-rpl32*, and *ccsA-ndhD* with similarity of 84.2%, 93.95%, and 94.83%, respectively (Table 1).

**Table 1:** Pairwise identity of IGS region among three complete cpDNAs of *Lilium lancifolium*.

Regions	Identity (%)		Regions	Identity (%)	
	KY748297-China	KY940844-Korea		KY748297-China	KY940844-Korea
*trnK_UUU-matK*	99.67	99.67	*psaI-ycf4*	100	99.72
*matK-trnK_UUU*	99.87	99.87	*ycf4-cemA*	100	99.86
*trnK_UUU-rps16*	99.73	99.46	***petA-psbJ***	**84**.**2**	**84**.**2**
*rps16-trnQ_UUG*	99.68	99.68	*psbE-petL*	100	99.92
*psbK-psbI*	99.2	99.2	*psaJ-rpl33*	99.6	100
*trnS_GCU-trnG_UCC*	99.71	99.71	*rpl33-rps18*	99.43	99.43
*trnR_UCU-atpA*	99.12	98.23	*rps18-rpl20*	100	99.63
*atpH-atpI*	99.89	99.89	*clpP* intron 1	99.87	99.75
*rps2-rpoC2*	100	98.28	*petB* intron	100	99.88
*rpoC1* intron	99.87	99.74	*rps11-rpl36*	100	99.24
*psbM-trnD_GUC*	99.92	100	*rps8-rpl14*	96.95	96.95
*trnE_UUC-trnT_GGU*	100	99.85	*rpl16 intron*	100	99.9
*trnT_GGU-trnE_UUC*	99.89	99.89	*ycf15-trnL_CAA*	100	99.86
*psaA-ycf3*	99.75	99.75	*rps7-rps12*	98.28	98.28
*ycf3* intron 2	100	99.02	*rps12-trnV_GAC*	99.95	100
*trnS_GGA-rps4*	99.65	99.65	*trnI_GAU* intron	100	99.89
*trnT_UGU-trnL_UAA*	99.87	100	***ndhF-rpl32***	**93**.**95**	**93**.**95**
*trnL_UAA* intron	100	99.26	***ccsA-ndhD***	**94**.**83**	**94**.**83**
*ndhC-trnV_UAC*	99.86	99.86	*psaC-ndhE*	99.75	99.75
*accD-psaI*	100	99.88	*rps15-ycf1*	99.51	99.51

The bold letters indicate regions which have low similarity (<95%).

### RNA editing sites and their potential effects

**Figure 2:**
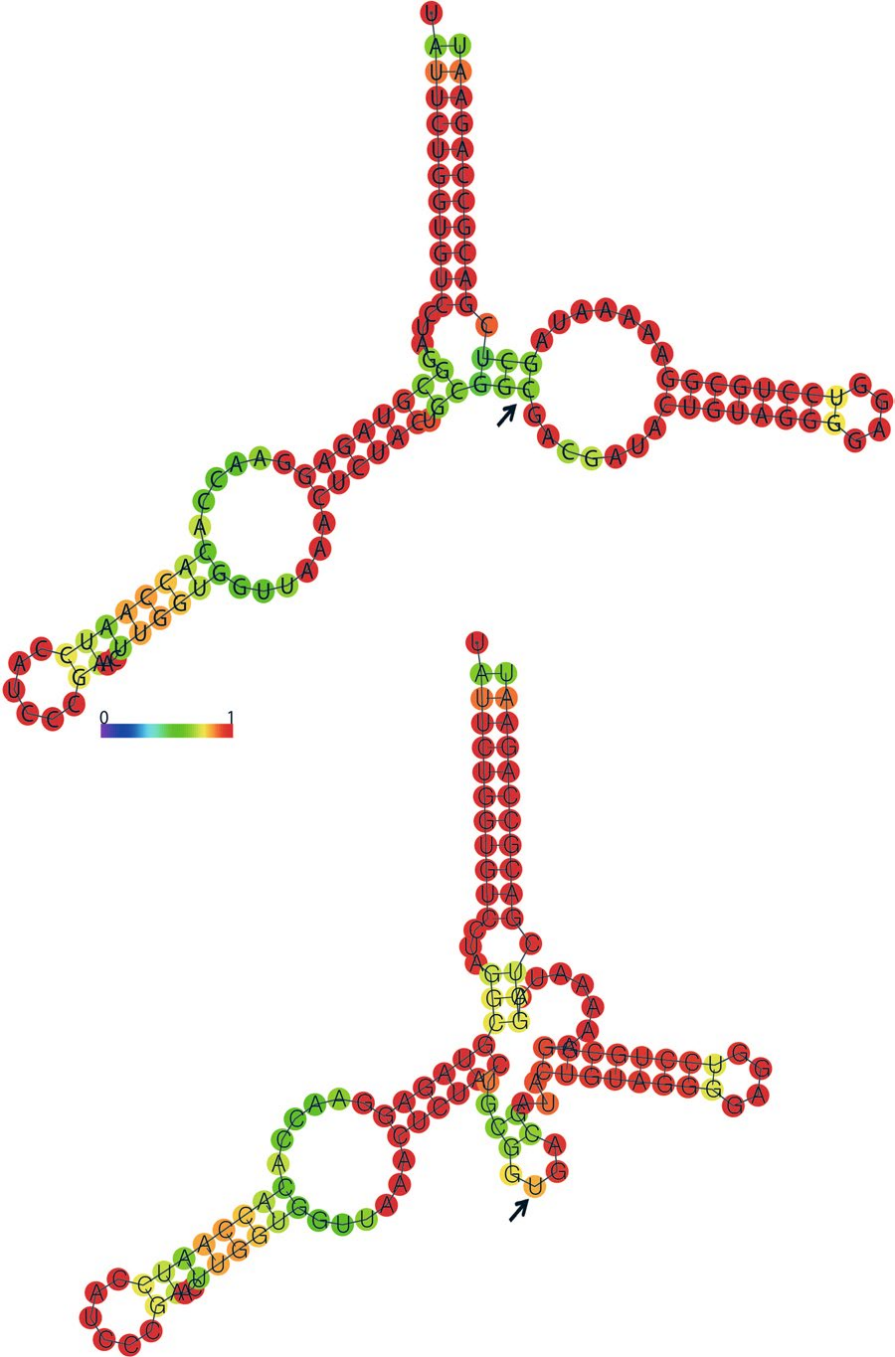
The predicted secondary structure of *rrn5S* with (lower) and without RNA editing site (upper). The color radian (from purple to red) means the probability of connection among nucleotides (from 0 to 1). The black arrows indicate the position of RNA editing.

The mapping results of transcription data to complete cpDNA of *L*. *lancifolium* revealed 90 editing sites, which located unequally among genes groups (Fig. 1B). The *ndh* genes possess the highest number of editing sites (29 sites) followed by the RNA polymerase genes (16 sites). The Rubisco gene (*rbcL*) has only one RNA editing site within its coding region. In contrast, *ndhB* gene has the highest number of editing sites (10 sites). The most abundant content of RNA editing in *L*. *lancifolium* is C-to-U conversion (Table 2). However, the U-to-C conversion was also found in *rpl36* and *rrn23*. Most RNA editing resulted in nonsynonymous substitution, of which the changes from S (Serine) to L (Leucine) is the most frequent (25 sites), followed by S (serine) to F (Phenylalanyl) with 17 sites. Nevertheless, 12 out of 90 editing sites resulted in synonymous substitution (Table 2). Additionally, a total of 32 editing sites were also found in IGS regions of *L*. *lancifolium* cpDNA (Supplementary Table 1). In comparison to the previous complete cpDNA of *L*. *lancifolium*, the *infA* and *cemA* were annotated as pseudogenes because of the presence of internal stop codons within the coding regions. However, the transcriptome data revealed that there are no significant RNA editing sites within the coding region of these two genes. The RNA editing occurred not only in protein-coding genes but also in *rRNA* (Table 2). Specifically, the U-to-C conversion was found in *rrn23S*, whereas the C-to-U conversion was recorded in *rrn5S*. A further check on the predicted structure of *rrn5S* showed that the editing event affected the structure of *rrn5S* (Fig. 2). The RNA expression level was also compared among protein-coding genes of *L*. *lancifolium* cpDNA (Table 3). The results showed that the *psbA* is the most expressed gene followed by *rbcL* and *petB*. Although the *ndh* genes have the highest number of RNA editing sites, their expression level is lower than other genes (Table 3). The *petL* has the lowest expression level.

**Table 2:** The number of RNA editing sites among coding regions of plastid genome of *L*. *lancifolium*.

Gene	Site	Position (aa/nucleotide)	Editing content	Coverage	Number of reads (percentage)
*rbcL*	1	50/150	P(cc**C**) → P(cc**U**)*	188389	C: 39 (0.02%); U: 187680 (99.62%)
*matK*	1	160/478	H(**C**au) → Y(**U**au)	751	C: 195 (26.1%); U: 556 (73.9%)
	2	245/734	S(u**C**u) → F(u**U**u)	3612	C: 1888 (52.3%); U: 1718 (47.6%)
*psbA*	1	232/696	S(uc**C**) → S(uc**U**)*	2011054	C: 387 (0.02%); U: 2002141 (99.56%)
*atpA*	1	258/774	S(u**C**a) → L(u**U**a)	2684	C: 37 (1.4%); U: 2646 (98.5%)
	2	383/1148	S(u**C**a) → L(u**U**a)	3878	C: 51 (1.3%); U: 3821 (98.5%)
*atpF*	1	31/92	P(c**C**a) → L(c**U**a)	3913	C: 331 (8.5%); U: 3579 (91.4%)
*atpI*	1	15/45	Y(ua**C**) → Y(ua**U**)	3682	C: 2736 (74.3%); U: 940 (25.5%)
	2	210/629	S(u**C**a) → L(u**U**a)	3580	C: 47 (1.3%); U: 3528 (98.5%)
*rpoC2*	1	1235/3704	S(u**C**a) → L(u**U**a)	493	C: 43 (8.7%); U: 451 (91.3%)
*rpoC1*	1	14/41	P(c**C**a) → L(c**U**a)	764	C: 203 (26.5%); U: 558 (72.9%)
	2	61/182	S(u**C**c) → F(u**U**c)	1004	C: 543 (54%); U: 462 (46%)
	3	107/321	I(au**C**) → I(au**U**)*	818	C: 487 (59.5%); U: 331 (40.4%)
	4	178/500	S(u**C**a) → L(u**U**a)	848	C: 98 (11.5%); U: 750 (88.3%)
	5	210/629	S(u**C**a) → L(u**U**a)	857	C: 149 (17.4%); U: 709 (82.6%)
	6	267/799	R(**C**gg) → W(**U**gg)	906	C: 43 (4.7%); U: 859 (94.7%)
*rpoB*	1	29-10	S(u**C**c) → F(u**U**c)	223	C: 81 (36.2%); U: 142 (63.4%)
	2	113/338	S(u**C**u) → F(u**U**u)	205	C: 82 (39.8%); U: 123 (59.7%)
	3	184/551	S(u**C**a) → F(u**U**a)	78	C: 66 (83.5%); U: 13 (16.5%)
	4	189/566	S(u**C**a) → F(u**U**a)	112	C: 54 (47.8%); U: 59 (52.2%)
	5	665/1994	S(u**C**a) → F(u**U**u)	233	C: 11 (4.7%); U: 223 (95.3%)
	6	807/2420	S(u**C**a) → F(u**U**a)	230	C: 31 (13.4%); U: 200 (86.6%)
	7	900/2698	P(**C**cu) → S(**U**cu)	286	C: 75(26.1%); U: 212 (73.9%)
*psbZ*	1	17/50	S(u**C**a) → L(u**U**a)	23558	C: 869 (3.7%); U: 22664 (96.2%)
	2	60/180	L(cu**C**) → L(cu**U**)*	15389	C: 14560 (94.6%); U: 810 (5.3%)
*rps14*	1	27/80	S(u**C**a) → L(u**U**a)	14988	C: 470 (3.1%); U: 14472 (96.6%)
*psaA*	1	51/153	A(gc**C**) → A(gc**U**)*	10434	C: 6 (0.1%); U: 10407 (99.7%)
*ycf3*	1	15/44	S(u**C**u) → F(u**U**u)	2070	C: 694 (33.5%); U: 1372 (66.2%)
	2	21/63	I(au**C**) → I(au**U**)*	1477	C: 640 (43.3%); U: 834 (56.4%)
	3	62/185	T(a**C**g) → M(a**U**g)	648	C: 502 (77.3%); U: 146 (22.5%)
	4	64/191	P(c**C**a) → L(c**U**a)	903	C: 423 (46.8%); U: 469 (51.9%)
*ndhJ*	1	43/128	S(u**C**a) → L(u**U**a)	968	C: 245 (25.3%); U: 724 (74.7%)
*ndhK*	1	23/69	P(cc**C**) → P(cc**U**)*	264	C: 155 (58.5%); U: 110 (41.5%)
	2	27/81	F(uu**C**) → F(uu**U**)*	343	C: 191 (55.5%); U: 153 (44.5%)
*ndhC*	1	13-5	H(**C**ac) → Y(**U**ac)	335	C: 69 (20.5%); U: 267 (79.5%)
	2	104/311	P(c**C**a) → L(c**U**a)	264	C: 155 (58.5%); U: 110 (41.5%)
	3	108/323	S(u**C**a) → L(u**U**a)	343	C: 191 (55.5%); U: 153 (44.5%)
*atpB*	1	395/1184	S(u**C**a) → L(u**U**a)	8704	C: 140 (1.6%); U: 8556 (98.3%)
*accD*	1	452/1355	S(u**C**a) → L(u**U**a)	625	C: 224 (35.8%); U: 393 (62.8%)
	2	466/1397	P(u**C**c) → L(u**U**c)	574	C: 245 (42.6%); U: 329 (57.2%)
*psaI*	1	25/74	S(u**C**u) → F(u**U**u)	1721	C: 579 (33.6%); U: 1140 (66.2%)
	2	27/80	H(**C**au) → Y(**U**au)	1152	C: 1078 (93.5%); U: 72 (6.2%)
	3	34/102	V(gu**C**) → V(gu**U**)	3243	C: 2758 (85%); U: 482 (14.9%)
*ycf4*	1	176/528	F(uu**C**) → F(uu**U**)*	2311	C: 1774 (76.7%); U: 538 (23.3%)
*psbJ*	1	20/59	P(c**C**u) → L(c**U**u)	21133	C: 392 (1.9%); U: 20673 (97.8%)
*psbF*	1	26/77	S(u**C**u) → F(u**U**u)	8436	C: 252 (3%); U: 8177 (96.9%)
*psbE*	1	72/214	P(**C**cu) → S(**U**cu)	13182	C: 145 (1.1%); U: 13023 (98.8%)
*petL*	1	2/5	S(u**C**u) → F(u**U**u)	2646	C: 559 (21.1%); U: 2084 (78.7%)
	2	19/56	P(c**C**a) → L(c**U**a)	1591	C: 48 (3%); U: 1540 (96.7%)
*rps18*	1	74/221	S(u**C**g) → L(u**U**g)	5264	C: 283 (5.4%); U: 4975 (94.5%)
*clpP*	1	26/82	H(**C**au) → Y(**U**au)	865	C: 110 (12.7%); U: 756 (87.3%)
	2	187/559	H(**C**au) → Y(**U**au)	1533	C: 107 (5.4%); U: 1402 (91.4%)
*psbN*	1	10/30	F(uu**C**) → F(uu**U**)*	57622	C: 12298 (21.3%); U: 45238 (78.5%)
*petB*	1	4/11	N(a**A**u) → S(a**G**u)	10646	A: 8356 (78.5%); G: 2268 (21.3%)
	2	142/424	R(**C**gg) → W(**U**gg)	27257	C: 290 (1.1%); U: 26917 (98.7%)
	3	206/617	P(c**C**a) → L(c**U**a)	9585	C: 220 (2.3%); U: 9351 (97.5%)
*petD*	1	162/484	Q(**C**aa) → stop(**U**aa)	13552	C: 248 (1.8%); U: 13286 (98%)
*rpoA*	1	67/200	S(u**C**u) → F(u**U**u)	947	C: 323 (34.1%); U: 622 (65.6%)
	2	123/368	S(u**C**a) → L(u**U**a)	1193	C: 254 (21.3%); U: 938 (78.6%)
*rpl36*	1	14-5	V(g**U**u) → A(g**C**u)	3479	U: 2 (0.1%); C: 3475 (99.8%)
*rps3*	1	157/470	T(a**C**a) → I(a**U**a)	1513	C: 74 (4.9%); U: 1440 (95.1%)
	2	195/583	H(**C**au) → Y(**U**au)	1951	C: 309 (15.8%); U: 1638 (83.9%)
*rpl2*	1	1/2	T(a**C**g) → M(a**U**g)	708	C: 340 (48%); U: 369 (52%)
*rpl23*	1	24/71	S(u**C**u) → F(u**U**u)	562	C: 84 (48%); U: 479 (85.1%)
*ndhB*	1	50/149	S(u**C**a) → L(u**U**a)	803	C: 88 (10.9%); U: 716 (89.1%)
	2	156/467	P(c**C**a) → L(c**U**a)	929	C: 41 (4.4%); U: 887 (95.4%)
	3	181/542	T(a**C**g) → M(a**U**g)	536	C: 55 (10.2%); U: 482 (89.8%)
	4	204/611	S(u**C**a) → L(u**U**a)	343	C: 58 (16.9%); U: 286 (83.1%)
	5	205/704	S(u**C**c) → F(u**U**c)	347	C: 53 (15.2%); U: 295 (84.8%)
	6	246/737	P(c**C**a) → L(c**U**a)	167	C: 65 (38.7%); U: 102 (60.7%)
	7	277/830	S(u**C**a) → L(u**U**a)	193	C: 123 (61.3%); U: 71 (36.4%)
	8	279/836	S(u**C**a) → L(u**U**a)	162	C: 80 (49.1%); U: 83 (50.9%)
	9	371/112	S(u**C**a) → L(u**U**a)	1134	C: 108 (9.5%); U: 1025 (90.3%)
	10	494/1481	P(c**C**a) → L(c**U**a)	1271	C: 188 (14.8%); U: 1082 (85.1%)
*ndhF*	1	21/62	S(u**C**a) → L(u**U**a)	674	C: 46 (6.8%); U: 628 (93%)
	2	87/259	H(**C**ac) → Y(**U**ac)	208	C: 51 (24.4%); U: 158 (75.6%)
	3	131/392	S(u**C**u) → F(u**U**u)	1205	C: 13 (11.1%); U: 1191 (98.8%)
*ccsA*	1	118/353	S(u**C**a) → L(u**U**a)	750	C: 70 (9.3%); U: 680 (90.5%)
	2	272/815	S(u**C**a) → L(u**U**a)	731	C: 68 (9.3%); U: 662 (90.4%)
*ndhD*	1	1/2	T(a**C**g) → M(a**U**g)	791	C: 285 (36%); U: 505 (63.8%)
	2	22/65	S(u**C**c) → F(u**U**c)	628	C: 82 (13%); U: 545 (86.6%)
	3	130/389	S(u**C**a) → L(u**U**a)	722	C: 129 (17.8%); U: 593 (82%)
	4	227/680	S(u**C**g) → L(u**U**g)	840	C: 190 (22.6%); U: 648 (77.1%)
	5	318/953	T(a**C**a) → I(a**U**a)	930	C: 124 (13.3%); U: 803 (86.3%)
*ndhG*	1	17/50	S(u**C**a) → L(u**U**a)	531	C: 115 (21.6%); U: 417 (78.4%)
	2	116/347	P(c**C**g) → L(c**U**g)	1198	C: 107 (8.9%); U: 1088 (90.7%)
*ndhA*	1	358/1073	S(u**C**c) → F(u**U**c)	1467	C: 198 (13.5%); U: 1226 (86.2%)
*ndhH*	1	30-10	L(cu**C**) → L(cu**U**)*	1107	C: 1020 (92.1%); U: 85 (7.7%)
	2	169/505	H(**C**au) → Y(**U**au)	740	C: 85 (11.5%); U: 651 (87.9%)
*rrn5S*	1	−/72	C → U	9350	C: 24 (0.3%); U: 9258 (99%)
*rrn23S*	1	−/1327	U → C	2532	C: 2148 (84.8%); U: 381 (15%)

The asterisk indicates synonymous substitution. Bold letters represent changes of nucleotides and their positions in the codons.

**Table 3:** RNA expression of protein-coding genes in the *L*. *lancifolium* chloroplast genome.

Gene	Length (bp)	RPKM	Gene	Length (bp)	RPKM	Gene	Length (bp)	RPKM
*psbA*	1032	557101	*atpI*	744	1798	*ndhK*	872	459
*rbcL*	1443	72754	*psbF*	120	1780	*ndhG*	534	454
*petB*	1467	11128	*atpF*	1338	1766	*rpoC1*	2837	451
*psbC*	1416	9863	*ycf4*	555	1700	*ndhB*	2215	425
*petD*	1233	9292	*cemA*	709	1460	*ndhD*	1506	411
*psbH*	222	8993	*rps11*	417	1391	*accD*	1470	400
*psbD*	1062	8199	*petA*	963	1363	*rps4*	606	382
*psbZ*	189	6822	*ndhE*	306	1341	*rps15*	273	369
*psaC*	246	6521	*ycf3*	1949	1259	*ndhH*	1182	365
*psbB*	1527	5815	*rpl14*	369	1235	*ccsA*	966	362
*psbN*	132	5486	*rpl16*	1420	1158	*rps12*	914	360
*psbE*	252	5439	*rps8*	399	1052	*rpl36*	114	338
*rps14*	303	5273	*infA*	228	1042	*rpl2*	1497	310
*psaA*	2253	4791	*ndhA*	2080	1004	*rps2*	711	303
*psaB*	2205	4244	*psbT*	102	932	*ndhC*	363	292
*psbJ*	123	3913	*psbI*	111	837	*rpl23*	282	220
*atpB*	1497	3584	*ndhJ*	477	827	*ycf1*	5577	207
*rpl33*	204	3280	*rps3*	657	788	*psaI*	105	190
*atpE*	408	3191	*rpoA*	1008	705	*rpl20*	354	177
*atpH*	246	2600	*ndhI*	540	680	*rpoC2*	4125	171
*psbL*	117	2587	*ndhF*	2229	641	*rpoB*	3207	133
*psaJ*	129	2369	*rpl32*	174	626	*psbM*	105	112
*psbK*	192	2181	*rps7*	468	622	*petN*	90	89
*rps18*	306	2058	*petG*	114	555	*ycf2*	6621	70
*atpA*	1524	1966	*rps19*	279	523	*ycf15*	231	56
*rps16*	1142	1964	*rpl22*	393	496	*petL*	960	13
*matK*	1539	1865	*clpP*	1991	488			

## Discussion

Previously, most of the plastid genome studies used the data of one individual as the representative of that species and focused on a comparative genomic analysis with closely related taxa^15,16,18^. This approach has not been fully providing detailed information on the diversification of cpDNAs within a species. Recently, Shi *et al*.^20^ reported 11 complete cpDNAs of both cultivated and wild watermelon. The cpDNAs of three individuals of each species were completed and compared to others. They showed that although the gene number and order are identical among examined species, the wild watermelon exhibited a significant change in the plastid genome size. In this study, the newly sequenced cpDNA revealed both conserved and diverse trends in comparison with previously published cpDNA of *L*. *lancifolium*. In fact, the cpDNA of *L*. *lancifolium* in this study is longer (105 bp) than those in previous studies which have an identical length (152,574 bp). Additionally, the Korean *L*. *lancifolium* has nonfunctional *cemA* which was found as a functional gene in Chinese counterpart. These findings suggested the interspecific diversification of plastid genome among wild plants and the need for further studies on this issue. Additionally, low similarities in IGS of *petA-psbJ*, *ndhF-rpl32*, and *ccsA-ndhD* suggest these regions as hot-spot sites for further studies on evolution of *L*. *lancifolium* and related species (Table 1).

RNA editing plays an important role during the post-transcriptional process because it alters the coding content of the genes by two pathways of insertion/deletion and conversion/substitution. Specifically, the C-to-U conversion altered a serin to phenylalanine codon in *psbF* mRNA of *Spirodela polyrhiza*^6^. Also, the formation of translation initiation, or internal stop codon, caused by RNA editing, was reported^9–11^. A similar trend was found in the transcriptome data of *L*. *lancifolium* (Table 2). The changes of amino acid composition among genes were mainly caused by C-to-U conversion. Also, the formation of the start codon in *rpl36* and *ndhD* genes resulted from C-to-U conversion at the second position in the start codon. In *L*. *lancifolium* cpDNA, *infA* and *cemA* genes were annotated as pseudogenes and expected to be corrected by RNA editing process. However, there are no RNA editing sites in mRNA of these two genes. Previously, the loss of *infA* in cpDNA was recorded in many plants^21^ and compensated by the nuclear *infA*. In fact, mRNA of nuclear *infA* was found by assembling transcriptome data to *infA* gene of *Pheonix dactylifera* (GenBank Accession XM_008784933). In contrast, the case of *cemA* needs further investigations. Among the four examined monocots, there are different numbers of RNA editing sites (Supplementary Table 2). Most of RNA editing sites resulted in nonsynonymous substitutions, except for *Phalaenopsis aphrodite* subp *formosana* of which more than half substitution is synonymous (Supplementary Table 2). However, most of the editing content is C to U in all examined taxa. Notably, the RNA editing in *Deschampsia antarctica* revealed the changes from G to A, G to C, A to G, A to C, A to U, and U to G^22^.

The transcriptome data revealed different expression level of genes in *L*. *lancifolium* cpDNA (Table 3). In comparison with RNA expression in *D*. *antarctica*, a member of the grass family, there are differences among expression level between *L*. *lancifolium* and *D*. *antarctica*^22^. One possible explanation could be the different habitat environment. In fact, *D*. *antarctica* adapted to the harsh environment of Antarctica whereas *L*. *lancifolium* distributes in temperate regions. Additionally, the gene expression is different during development stages of plants. In this study, we used only the leaf tissue at the growth stage of *L*. *lancifolium*. Therefore, further studies of transcriptome of various tissues at different development stages should be conducted to explore the overall trend of expression in *L*. *lancifolium*.

Previously, Chen *et al*.^7^ reported the effect of RNA editing on stabilizing the secondary structure of *trnM* in *Phalaenopsis aphrodite* subsp f*ormosana*. In this study, RNA editing site resulted in changes in the secondary structure of *rrn5S*. These data revealed the effect of RNA editing process on the stability of rRNA and tRNA. Although RNA editing sites have been recorded in protein coding regions of plastid genomes, studies on the secondary structure of these changes have not been fully conducted. Therefore, further investigations should be done to give a deeper understanding of this issue.

To sum up, the first plastid genome analysis of *L*. *lancifoliun* provided fundamental information for further studies on post-transcription events in Liliales. In fact, the RNA editing process is not able to reverse the pseudogenization caused by genomic events in plastid genomes of *L*. *lancifolium*. In this study, only leaf tissue was used for plastid transcriptome study, which does not fully reflect the evolutionary information in *L*. *lancifolium*. Therefore, transcriptome data of other tissue should be generated to trace the evolutionary history in *L*. *lancifolium* and other species of Liliales.

## Materials and Methods

### Plant materials, total DNA extraction, and RNA isolation

The mature fresh leaves of *Lilium lancifolium* during the growth stage were collected in its wild habitats. The specimen of *L*. *lancifolium* was made and deposited to Gachon University Herbarium. For DNA extraction, the fresh leaves were dried in silica gel before extraction steps with Plant DNeasy Mini Kit (Qiagen, Korea). To isolate total RNA, the fresh leaves were immediately put in liquid nitrogen after collected. Then, they were stored in the cold condition until being used for RNA isolation, which was conducted using Plant RNeasy mini Kit (Qiagen, Korea). Both DNA extraction and RNA isolation were conducted based on manufacturer’s instruction. The quality of DNA and RNA were tested using gel electrophoresis and one spectrophotometer.

### NGS generation, genome assembly, RNA editing determination and prediction of rRNA structure

To generate NGS data, the total DNA and RNA from leaves of *L*. *lancifolium* were applied to Illumina Hiseq 200 and Nextseq 500, respectively. First of all, the DNA and RNA were fragmented. Then, newly fragmented DNA and RNA were hybridized and ligated with adapters. In the next step, PCR amplification was employed to create the sequence library. Finally, the library was sequenced and resulted in the DNA-NGS data of 301 bp in length and transcriptome data of 76 bp in length. The DNA-NGS data were imported to Geneious program for further analysis^23^. The reads were trimmed with more than 5% chance of an error per base before being assembled to reference cpDNA of *Lilium lancifolium* (Accession number KY940844) with similarity over 95% between reads and reference sequence. Consequently, there are 3,852,736 reads of which 17,473 reads (0.45%) were assembled to reference with coverage of 34.5x. The newly completed cpDNA of *L*. *lancifolium* was annotated and manual adjusted in Geneious program. The map of cpDNA was illustrated by OGDraw^24^ with manual modification. The new cpDNA in this study was aligned with previously reported cpDNA of *L*. *lancifolium* (Accession number KY748297 and KY940844) using MAUVE alignment embedded in Geneious to identify hot-spot regions^25^. Also, the newly assembled cpDNA (GenBank Accession number MH177880) was used for identifying RNA editing sites. The RNA sequence data were imported to Geneious and aligned to cpDNA of *L*. *lancifolium* using Bowtie 2.0 with mismatch ≤2^26^. The filtered reads (26,824,116 out of 53,643,506 reads) were then analyzed using Cufflinks to calculate the read per kilobase million (RFKM) and TopHat for variants calling^27^. The determination of RNA editing sites was based on the division of reads with editing based on the total reads of that position. If the frequency of C-to-U or U-to-C conversion was over 5%, that position was recognized as an RNA editing sites as described in previous study^28^. An online tool (http://rna.tbi.univie.ac.at/) was used to predict the second structure of rRNA^29^. Transcriptome data of *L*. *lancifolium* was deposited to NCBI (SRA accession SAMN08940087).

## Supplementary information

Supplementary Tables
